# African Swine Fever: A One Health Perspective and Global Challenges

**DOI:** 10.3390/ani15070928

**Published:** 2025-03-24

**Authors:** Arianna Ceruti, Rea Maja Kobialka, Ahmed Abd El Wahed, Uwe Truyen

**Affiliations:** Institute of Animal Hygiene and Veterinary Public Health, Leipzig University, 04103 Leipzig, Germany; rea_maja.kobialka@uni-leipzig.de (R.M.K.); ahmed.abd_el_wahed@uni-leipzig.de (A.A.E.W.); truyen@vetmed.uni-leipzig.de (U.T.)

**Keywords:** African swine fever, one health, transboundary animal diseases

## Abstract

African Swine Fever is non-zoonotic viral disease affecting pigs worldwide, with severe implications for many sectors such as national economies and pig populations. The disease control strategies are variable across the globe and its implementation and outcome depend on many factors. One Health describes the collaborative effort across disciplines to address public health challenges involving human, animal, and environmental aspects. African Swine Fever is not commonly understood under the concept of One Health. However, the disease has a great impact on all One Health interfaces. This paper reviews and discusses how the vast impact of ASF, a non-zoonotic disease, still demands a holistic perspective to address global challenges and opportunities to control the disease.

## Simple Summary

African Swine Fever (ASF) is a devastating viral disease affecting domestic and wild pigs globally. Though non-zoonotic, ASF demands a One Health approach due to its far-reaching impacts across animal, human, and environmental interfaces. For animals, ASF causes high mortality in pig populations, with various clinical presentations depending on strain virulence. Control measures like culling further increase animal losses and raise ethical concerns. Environmentally, ASF impacts wildlife conservation through fencing measures that restrict movement of multiple species. Control efforts targeting wild boar populations disrupt ecosystems, while threatening endangered swine species globally. For humans, ASF severely impacts food security and economies. China alone suffered approximately $111 billion in losses. Small-scale farmers face existential threats, leading to poverty and non-compliance with control measures. Successful control strategies require context-specific approaches. The FAO/WOAH Global Framework for Transboundary Animal Diseases provides guidance, but implementation must be tailored to local contexts. ASF control demands interdisciplinary collaboration through public-private partnerships and transnational cooperation. Effective policies must balance disease control with economic sustainability, recognizing that ASF reflects the complex challenges of our globalized world.

## 1. Introduction

African swine fever (ASF) is a devastating and highly infectious viral disease affecting domestic and wild pigs which has impacted swine production systems throughout the world. Due to its massive implications for national and international economies, veterinary services as well as animal welfare policies, this transboundary animal disease (TAD) is of great significance. Consequently, it is listed as notifiable to the World Organisation for Animal Health (WOAH, founded as Office International des Epizooties, OIE). The causative agent, African Swine Fever Virus (ASFV), is a complex non-zoonotic pathogen, that challenges animal health, human interactions, and environmental implications. Since 2005 the disease has spread to 74 countries [1], and 5 continents in over a century [2].

The modern understanding of One Health reached the international stage with the Avian influenza crisis in the mid-2000s, highlighting the demand for interdisciplinary and integrated health approaches [3]. The complex interlinked system between animals, humans, and the environment demands a holistic perspective [4]. Food security and safety, climate change, and antibiotic resistance are major challenges, cutting across all three domains [5,6]. Interdisciplinary researchers, policymakers, the general public, and clinical practitioners are needed as key players. Zoonoses, such as avian Influenza, are often depicted as the most obvious One Health linkage, where pathogens are transmitted from animals to humans [7]. However, One Health could allow a deeper view into the ramified framework that comes with the circulation of a non-zoonotic pathogen such as ASFV.

This review aims to highlight the importance of ASF in a global and local context and the implications for animal, human, and environmental systems.

## 2. ASF at the Animal Interface

African Swine Fever Virus (ASFV) is a complex dsDNA virus causing the disease of African Swine Fever. ASFV has a narrow host range. It includes the Suidae family and the soft tick genus *Ornithodoros* as arthropod vectors [8]. As of now, there have not been any reported instances of the agent having zoonotic potential. This could be attributed to the absence of related viruses in human hosts that could serve as partners for recombination, as well as the low rates of recombination and mutation, due to the precise DNA proofreading polymerase [9].

The disease can affect all ages and both sexes in swine herds and the clinical picture of ASF can be divided into five forms, and are mainly associated with strain virulence: peracute, acute, subacute, chronic, and asymptomatic (Table 1). Other factors affecting the clinical course of ASF are the route of infection, individual host background, infection dose as well as the endemic status of the affected area. A definite clinical picture is not found in the case of infection with ASFV. An afflicted animal may exhibit various clinical signs, particularly during the initial phases of the illness. Hence, only a laboratory diagnosis can provide conclusive evidence. Morbidity levels within the herd may differ significantly based on factors such as the species of swine, the type of pig production system, management practices, and the level of biosecurity measures implemented. Despite its high lethality among susceptible pig populations, ASF does not spread as easily as foot-and-mouth disease or classical swine fever [10,11]. Case fatality in the first two weeks is high, however, contagiousness of the disease over time is relatively low and thus disease spread is often slow [12]. These epidemiological traits are unique to ASFV and should be considered when planning and implementing control measures.

**Figure 1:**
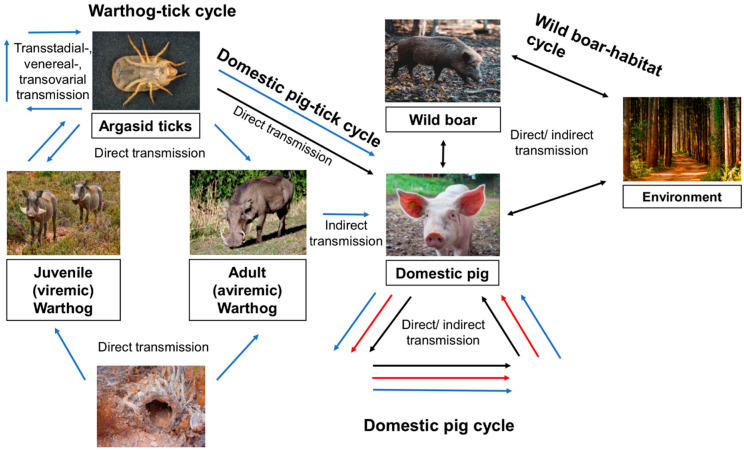
Various transmission cycles of African Swine Fever (ASF) and the interplay among its agents are observed. Blue arrows delineate transmission routes in Africa, black arrows those in Europe, and red arrows those in Asia.

The transmission cycle of ASF reflects the vast interlaces between human, animal, and environmental interface. ASF directly affects wildlife, in particular wild boars, as they are involved as disease hosts. Furthermore, the geographical distribution of hosts, climate, pig production system, and human interaction all impact disease transmission. Therefore, the transmission pattern is different in Asia, Europe, and Africa as illustrated in Figure 1.

The burden on swine populations is further increased by culling measures of infected and non-infected herds. This intervention is a widely implemented measure against ASF spread and leads to major animal losses [17,18]. After the first 33 ASF outbreaks in China, around 50,000 infected and affected pigs were culled [19]. In Vietnam, about 40% of the pig population of heavily affected areas was lost. Around 5 million pigs were culled in China [20]. Ethical questions are raised by these control actions and are often rejected by farmers and public opinion [21,22]. They are, however, usually outweighed by the expected positive economic effects for ASF spread control. This drastic action should be carefully implemented according to each epidemiological situation, using clear scientific communication and recognizing the ethical dilemma behind the action [23]. This dilemma exposes the assumption that domestic pigs are worth less than economic profit and juxtaposes the speciesism view against the anthropocentric view on ASF management. These questions should be discussed and kept in mind when addressing ASF in a holistic approach.

## 3. ASF at the Environmental Interface

The environment is often the most underrepresented pillar of One Health [24]. Nonetheless, ASF impacts the environment in various ways. In 2004, the Wildlife Conservation Society recognized the link between human and animal health and the threats of disease spreading that derive from it. Here, particular focus was put on the inclusion of wildlife health as an essential component of global disease prevention, surveillance, and control [25]. The disease could have indirect and direct negative impacts on various environmental scales: from wildlife conservation to climate change.

When looking at the broad environmental picture, it is difficult to put climate change into relation to ASF, and vice versa. The impact of climatic factors on disease dynamics is unclear since studies on possible indicators are still lacking. The first data, however, indicates that future climatic conditions will be favorable for wild ASFV spread [26]. Climatic factors could theoretically have effects on ASF indirectly through modification of wild boar and human movements, plant growth, and viral persistence in the environment [27]. Here, further research is needed. Not only does climate indirectly affect the spread of ASF, but the disease also impacts the climate. The disease presence in various countries affects greenhouse gases and climate change by disrupting the pork meat supply chain. Due to ASF, animal losses are vast across countries, and this leads to greenhouse gas emissions which ultimately do not translate into the production of pork meat. A modelling exercise showed that around 45 million tonnes of Greenhouse gas emissions invested in producing pigs did not reach the global food supply chain due to ASF. That equals to average annual emissions of 16.7–25.1 million cars in Germany [28]. More data on the effects of pig health on environmental sustainability and climate change is needed to stress this issue and further complement policy actions regarding ASF.

Zooming in, ASF can have direct harmful impact on wild pigs across the globe. The Eurasian wild pigs, i.e., feral pigs (*Sus scrofa ferus*) and wild boars (*Sus scrofa domesticus*), play a pivotal role in European transmission cycle, where genotype II has been circulating since 2007 [29]. This cycle was first described when ASF reached the Spanish peninsula in the 1960s, which is a natural habit for Sus scrofa. After 2007, this pattern was further reported in Central and Eastern Europe, where wild boars are widely present and freely move through borders. Here, it was reported that Eurasian wild boars, which are densely present, especially in Poland and the Baltic States, were able to maintain ASFV without the need for domestic pigs to reintroduce the virus [12]. Various *Ornithodoros* tick species circulate in Mediterranean regions, while it is unlikely that these contributed substantially to the ASF spread and persistence in these regions [30].

The disease has various impacts on wildlife conservation. ASF poses not only a threat to wild and domestic pigs and warthogs but also to other endemic and possibly endangered swine species around the globe, such as the Visayan warty pig or Pygmy hog [31]. In European countries, intensive hunting of wild boar in protected areas is used as a measure to slow ASF spread. This approach of reducing potential ASF hosts is, however, controversially discussed. Animal defenders protested actively against culling measures of wild boar in some countries [21,32]. Additionally, it was pointed out early on by veterinarians to still follow animal welfare laws when intensively hunting wild boar [33]. For instance, only about 10–22% additional mortality could be achieved in an already infected area with ASFV through active wild boar hunting [34].

Other wildlife, such as deer and wolves, also suffer indirectly from the disease control measures. A key aspect of ASF prevention and control in the wild boar population that is used in most European countries is the fencing around the outbreak area. Three distinct areas are determined: an infected area, a buffer zone (both surrounded by a full fence), and a surveillance zone [35]. Different types and lengths of fencing are used to minimize and hinder wild boar migration. In German states, permanent fences are used. For instance, in Lower Saxony, it covered 150 km, and in Brandenburg 532 km with an additional 117 km of electric/site fence [36]. However, from an environmental point of view, this fence poses difficulty to other wildlife and has been actively pointed out by environmentalists [37]. Certain fence locations can be problematic by blocking animals from leaving a floodplain area that is regularly flooded. According to one report, up to 19 deer were found dead right at a fence in Brandenburg after intense flooding [38]. It can be assumed that smaller wildlife is also hindered by the trespassing of fences. Small passages and ramps along the fence are mentioned as a possible solution [39]. However, an improvement of this situation through these measures is often unclear. They compromise the effectiveness of the fence against ASF. All in all, there are long-lasting negative implications for wildlife through ASF outbreak management. Nonetheless, fencing might be a reasonable and temporary solution for certain disease incursion areas. In South Korea, for instance, biosafety regulations were not put in place appropriately when applying ASF control measures to wild boar. Accordingly, trapping (with appropriate biosecurity) and fencing are postulated as the most effective approach in this specific context [40]. From a solely virological approach, fencing is drastic but effective in ASF-free countries or recently affected areas since it slows down viral spread. Nonetheless, approaches and perspectives used for ASF should be adapted over time according to the dynamics of the disease in each region. Existing methods can be overwritten or complemented. Additionally, alternative preventive methods, such as the genetic resistance of domestic pigs to ASF, could become viable options in the future [41].

These environmental issues, adding to the economic implications of fencing, need to be considered and put into perspective when disease control measures against ASFV in wild boar populations are to be established.

## 4. ASF at the Human Interface

Food and agricultural sectors are essential to cover the supply demands of the rapidly increasing world population. Agricultural productivity, including meat production, is necessary to cover high-quality protein intake. The WOAH states that more than 70% additional animal protein will be needed to feed the world by 2050. At the same time, 20% of global food production loss is caused by diseases [42].

In developed countries, large-scale production systems have achieved high levels of performance. For instance, the European Union (EU) is the largest pig meat exporter. This was also enhanced by the pig production fall in Asia due to ASF [43]. Small pig holdings have decreased in the last decade, enhanced by the presence of ASF since 2014 [44]. In developing countries, small-scale production systems or backyard farming are the most common forms of pig production [45,46]. During the past three decades, however, pig production has changed rapidly from smallholders to an intensive industry in many developing countries. Every type of farming is affected by ASF, leading to consequences not only for animals but also for their owners.

Bigger production systems tend to have higher financial security. However, ASF restrictions affect the whole country’s pork export and trade regulations. Trading limitations harm food security and the agricultural sector [47]. Vietnam reported a 13.8% reduction in pork production due to the ASF outbreak in 2019, while Chinese pork production dropped to around 55%, leading to an estimated $111.2 billion loss in that year [48,49]. Estimations about the costs of ASF epidemics in the Philippines (2019) and Lao Cai, Vietnam (2020) ranged widely from $8 million in Vietnam to more than $58 million in the Philippines [50]. A literature review estimated the range of economic losses for ASF from $649,000 to $94 billion (2019) [51]. Due to the huge disruption of Chinese pork production, the fluctuation of pork prices was immense, leading to an increase of 47% [52]. A positive effect of the ASF-related increase in pork product prices was reflected in a growing alternative protein-supplying market, such as beef, chicken, and aquaculture [48].

On a smaller scale, farmers’ and animal holders’ existence is directly threatened by the ASF pandemic. In Africa and Asia, the most common form is small-scale backyard farming. As a result, over 70% of all ASF outbreaks in Asia were detected in small herds [48]. Existential and financial fear can lead to a lack of farmers’ compliance with ASF regulations. This might induce a counterproductive effect, where a quick pig movement and slaughter are pursued to avoid economic loss while driving the disease’s spread [41]. This impact of the disease promotes poverty in these regions. In Chinese culture, for instance, pork is a popular food source and indicates the family’s financial status. Thus, pig production and pork prices influence directly people’s livelihood stability [53]. In addition, the stigmatization of animal holders of an ASF-infected herd is a negative side effect that should not be underestimated [54]. Compliance of farmers and communities is essential for adequate outbreak response. Therefore, community engagement needs to be an integral part of field outbreak investigations. The deployment of interdisciplinary One Health rapid response teams could bridge the gap between animal holders and national authorities [55,56].

The difficulties of local disease control policies are clearly shown when an ASF outbreak response and control is put into place. Enhanced biosecurity, disease tracking, extensive disinfection, movement restriction (of animals, people, and products), and possible preventive culling, are some of them [57,58]. In Asia, almost 5 million pigs died or were culled because of ASF as of August 2019 [59]. In contrast, no eradication policies also have vast consequences. Spain’s no-eradication policy between 1960 and 1985 led to loss of reportedly 3.5 million naïve pigs due to the disease and a trading ban of around 30 years [60,61].

From a socioeconomic and animal welfare perspective, culling is a drastic measure that must be modulated depending on the local outbreak. Here, mental distress can be assumed when culling is imposed on a pig holder and veterinarians implementing the measure, as already described for depopulation measures against other animal diseases [62,63]. Control measures such as culling are addressed to animals and have many implications. However, global ASF spread is mainly human-driven and this needs to be taken into consideration.

Thus, the outlined changes and impacts in the food chain system are diverse and need to be considered when applying local disease control measures. A One Health approach can help prevent ASF spread and its implications [64,65].

## 5. Challenges and Opportunities

### 5.1. Globalisation as a Key Disease Driving Factor

The disease was first reported in domestic pigs in Kenya in 1921 [66]. Due to the importation of European domestic pigs into Africa shortly before the first outbreak, the balance of the ancient natural cycle between hosts and the causative agent was disrupted [67,68]. By the end of the 1950s, ASF was found in most countries in Eastern, Southern, and Central Africa [69,70]. The virus then spread to Western Africa [71]. The first international case in Europe was reported in 1957 in Portugal, probably due to the importation of infected pork products fed as swill [72]. Over 50 years (1957–2007), the virus appeared in Central American countries, Brazil [69,73], and in several European countries [74]. All non-African regions, except Sardinia, managed to eradicate ASF by depopulation until the turn of the century [57]. In sub-Saharan African countries, ASF is still endemic [75].

**Figure 2:**
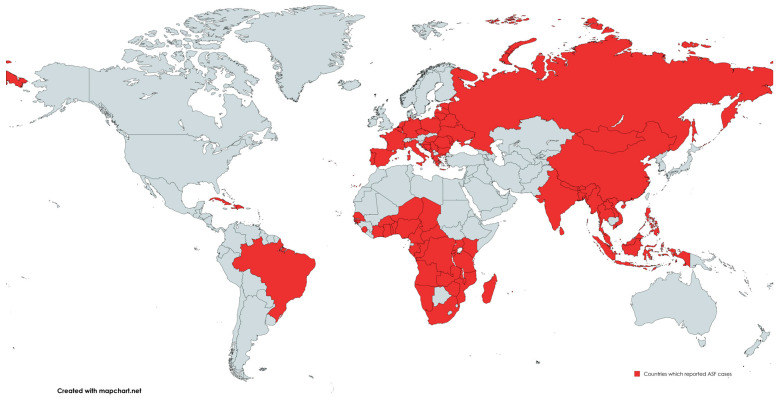
Countries where ASF outbreaks have been confirmed in wild boar or domestic pigs since the agent’s identification. Information was collected from WAHIS [80] and CISA-INIA [81]. Accessed on: 7 March 2025.

With growing globalization, increasing demand for pork, and the still very diverse biosecurity measures and outbreak response systems, the disease reappeared in 2007 in the Republic of Georgia [76], steadily spreading to other Transcaucasian countries, the Baltic States and Russia [77]. Upon the arrival of the virus to China in 2018 [78], the disease made the jump to the Asian continent, where it spread rapidly, even reaching remote regions such as Papua New Guinea [79]. So far, only the continent of North America has not reported an ASF case (Figure 2).

Since the early 20th century, there has been an increased circulation of people, animals, goods, and services. This has led to the spread of infectious diseases like ASF across borders via land, air, and maritime routes [82]. However, even non-affected countries can suffer consequences from ASF outbreaks by serious trade limitations. Not only the direct pork trade was severely disrupted. Other markets, such as the world oilseed trade, had to deal with its indirect consequences. China is the main importer and consumer of Soybeans. Since ASF affected its huge pig industry, the Chinese soybean demand was drastically lowered [52]. The biggest suppliers Brazil, the United States, and Argentina saw their soybean exports hindered. Oilseed prices were expected to remain low [83]. This example shows how international markets are interconnected. The problem of sustainability of the global food supply chain is highlighted by ASF outbreaks across countries. This stresses a demand for international collaboration and coordination, ultimately leading to an integrated approach in the local and regional context to mitigate trade fluctuations.

A benefit from globalization, however, is the increased exchange of knowledge and ideas, due to the connectivity across the world. The societal challenge of ASF can be confronted with shared experiences. Networks, research cooperations, and animal practitioners join forces to address ASF at each level. Resilience, unity, and vision are needed and can be achieved both regionally and internationally with engaged stakeholders across sectors.

### 5.2. New Preventive Methods to Mitigate ASF Impact

Different interventional preventive methods against ASF are currently being explored and could complement classical control measures.

To tackle wild boar depopulation and therefore reducing the diseases spread, approaches such as poisoning or immunocontraception have been discussed. Poisoning is illegal in EU and could pose great harm potential for the environment and other wildlife. However, in other Asian countries it is accepted and being implemented [84]. Other methods such as immunocontraception could be another solution, but it is still in a very early research stage and is not yet feasible [34].

Vaccination campaigns are an essential approach to mitigate infectious disease spread and clinical outcomes [85]. To date, there is no globally available vaccine for ASF. After the successful production of ASF vaccines, scalability and fair vaccine delivery to countries all around the world will be problems to address with fair policymaking. The COVID-19 pandemic impressively highlighted the potential of vaccine development, but also the issues of scalable vaccine production and its equal delivery around the world [86].

Different approaches are used for vaccine development. The commonly used approach of inactivated vaccines was shown to be non-protective for ASF, probability due to its incapability to induce cellular immune responses [87]. Vectored and ASF subunit vaccines are safer than live-attenuated vaccines (LAVs) and allow differentiation of infected from vaccinated animals (DIVA). Nonetheless, they only appear to confer partial protection and are not yet applicable. Additionally, subunit vaccines could not be used for oral vaccination of wild boar, since it requires a live vaccine [88]. LAVs for ASF have been shown to provide homologous protection, however with safety concerns and undesired complications [89]. Nonetheless, the LAV FOR ASFV-G-ΔI177L in Vietnam is the first commercial ASF vaccine in the world, but its use is limited to the country [90]. The development of LAVs is characterized by high costs and long cycles, which limit their rapid development.

Once ASF vaccination is marketable, it should always be embedded in classical prevention and control strategies, both for wild boar herds and domestic pig populations [9]. Appropriate vaccination strategies are an important asset for disease control. However, strategies will have to be determined according to each target swine population and socioeconomic factors.

### 5.3. The Need for Specific One Health-Oriented Action Plans

**Figure 3:**
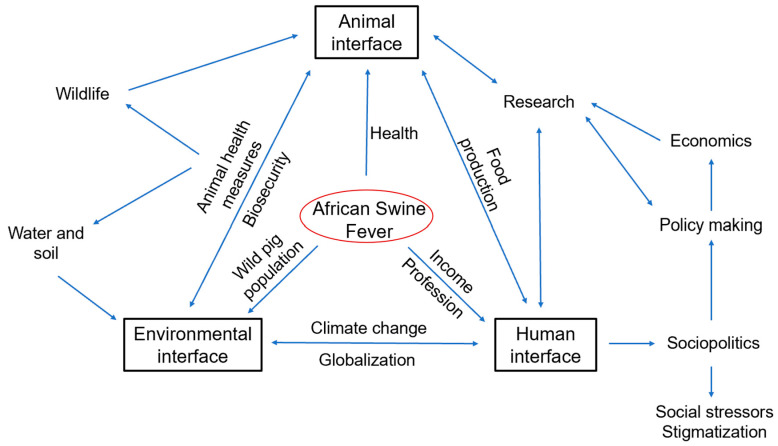
Schematic representation of ASF implications at the three One Health interfaces. Blue arrows indicate the interactions between the various interfaces.

As depicted in the last sections, ASF impacts animal-, human- and environmental interfaces in many ways (Figure 3). Specific and context-driven action plans could offer advantages in disease mitigation and control.

**Figure 4:**
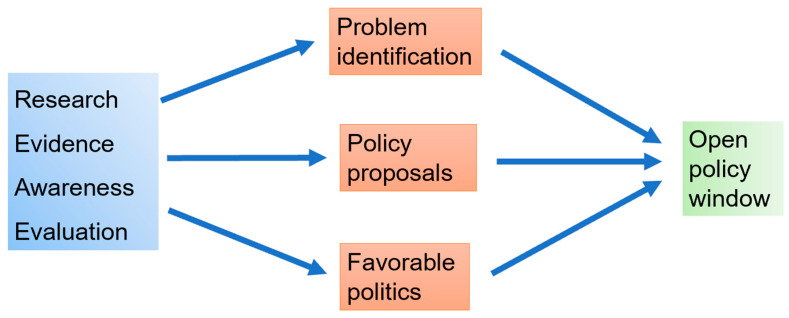
The Kingdon’s process streams leads to the open policy window. This process elevates societal problems, such as the ASF epidemics, to the political agenda and leads to necessary actions [92]. Blue arrows indicate interdependency.

A Global Framework was developed by FAO/WOAH to tackle Transboundary Animal Diseases (GF-TADs). Within this, a special initiative was published for ASF, outlying the factors and principles for successful global disease control [91]. The objectives include improvement of the national capabilities to control ASF using WOAH standards that are based on the latest science, establishing an effective coordination and cooperation framework for the global control of ASF, and easing business continuity. This framework sets an important stone to address the issues in an international context. Putting theory into practice, however, is the most challenging part when translating a framework to the national level. Clearly defined problems regarding ASF, feasible policy options, and politics merge into a so-called open policy window (Figure 4) [92]. This window of opportunity stresses a solution for ASF-related challenges. Current ASF research and awareness in a specific region influence ASF related problems, for example the first notification of the disease in a need country. Accordingly, policy proposals are issued, e.g., meat import-export restrictions. Ultimately, favorable local and regional politics are needed to successfully implement the policies, using the open policy window. However, the specific variables are different in each country. Prevention and control are heterogeneous depending on the national and regional context. ASF spread relies on various drivers, such as pig production practices and its wildlife interface, socio-cultural habits, animal health infrastructure as well as a governance framework.

Preventing the introduction of the disease or its spread in new countries is generally based on social awareness, farm-level biosecurity measures, national border inspection, and the integration of the socioeconomic situation in policy making. Another key aspect is surveillance for early detection of ASF. Nonetheless, the surveillance system needs to be risk-based tailored, and consider the social context to achieve optimal results with available resources [93]. The system may include passive and active surveillance. Passive surveillance is considered a key component of ASF control [17,94,95]. It can be enhanced with different incentives and awareness campaigns, leading to more compliance from all involved actors and possible observers [96]. Active disease monitoring is based on viral or serological ASF detection and needs to be adapted to the local context [97].

In the United States, the majority of the 65 million pigs are raised in high-biosecurity facilities [98]. The main disease introduction risk routes are considered to be the movement of live animals or their products, animal feed, or intentional viral spread (bioterrorism) [99]. Well-established control strategies are outlined by the Foreign Animal Disease Preparedness and Response Plan [100]. A potential ASF outbreak is estimated to cost $80 billion and would demand a full response plan to be put into place to contain the disease [101]. To this date, passive surveillance is being conducted in the USA [102,103]. Additionally, risk communication and diagnostic capacities are present [99].

Exceptionless stamping out actions are engaged through Russian and Chinese policies [35]. In other Asian countries, for instance, in the socialist republic of Vietnam, partial stamping out strategies are used. ASFV negative-tested pigs from an outbreak farm are allowed for slaughter and human consumption, saving resources and reducing the environmental impact [104]. In South Korea, besides strict movement control, special emphasis was given to quarantine measures at airports and ports even two years before the first outbreak, investigations in farms that used swill as pig feed, and increased awareness campaigns [105].

In the African continent, ASF policies are also very variable between countries. Not only different transmission routes are found [106], but also factors such as lack of awareness, underreporting, unregular stamping out without appropriate compensation, and fewer lab diagnostic capacities hinder sustainable disease control [107]. Predominantly small-scale extensive pig production is found, with South Africa, Nigeria, and Uganda as the main producers. South Africa, for instance, allows specific quarantine and culling measures within different compartments in the affected area [108]. The International Livestock Research Institute, together with the WOAH, stated that there is a general lack of intra-regional cooperation in Africa for ASF [109]. An action plan for the implementation of the regional strategy control of ASF in Africa has been developed and needs to be put into practice [110].

Within the borders of the EU, tackling ASF has been a major challenge since 2007. Wild boars serve as a viral reservoir and are a key element for its spread within European regions and the resulting loss of ASF-free status. Although the cross-border spread is relatively slow [111], the disease can eventually spill over to domestic pig populations. For wild boar control, the principle of regionalization and the respective measures to be implemented are pivotal. Eradication attempts for ASF in an affected country are always difficult and costly, and sometimes long-lasting over decades. For example, during Spain’s endemicity of ASF, the country was able to implement a structured and externally funded eradication plan only in 1985, almost three decades after its initial detection and unsuccessful previous measures. A central aspect for the successful ASF elimination after the program’s start was the close collaboration between the Spanish autonomous communities and government, the Portuguese government and the European Union [112].

Eradication success depends on different factors such as the ecological, epidemiological, and sociopolitical context. Therefore, ASF management requires great effort and collaboration between farmers, public opinion, policymakers, and veterinary authorities to address the socioeconomic challenge of ASF. One example of effective control strategies is shown by the Czech Republic. ASFV was first reported summer of 2017 and declared eradicated 19 months later. An essential factor was its early nationwide passive surveillance strategy of all found carcasses since 2014 [113]. The central approach was the zone demarcation management, which included an infected area (restriction to the public, fences, intense carcass surveillance, and fast removal), a buffer zone (ban of the measures mentioned in the buffer zone, to avoid the wider spread of animals) and a control zone (strict depopulation policy). In addition, these aspects needed the compliance of relevant authorities and hunters, achieved through enhanced awareness and education as well as financial motivation. An EU regionalization via zoning ensures not only strict measures to be applied but also a continuous safe trade of pork products from non-affected areas. Additionally, country-wide trade banning does not align with the World Trade Organization Agreement on the Application of Sanitary and Phytosanitary Measures [114]. However, even implementation measures such as zoning should lead to effective disease control while mitigating the economic loss at the regional level. Holistic policies based on updated epidemiological evidence could offer advantages for the human interface. For instance, a differentiated approach to ASF outbreaks in wild boar and domestic pig populations. Currently, the confirmation of an ASFV-positive animal, domestic or wild, leads to the implementation of strict policies and trade bans for the affected region. However, two different epidemiological scenarios can be understood. Wild boar populations in Eastern Europe are regularly tested positive due to the widespread natural habitat and the agent’s endemic circulation. A positive wild boar population does not have necessarily a direct impact on the quality of the local pig production chain. ASFV-positive domestic pigs around the affected wild boar population are sporadic and would have, in contrast, direct consequences. By implementing a mitigated trading ban policy on negative domestic pig farms in positively tested wild boar areas, the local value chain could be maintained and the economic loss reduced.

Disease control measures need to be sustainable. They require an agreement between inter-sectoral and trans-disciplinary stakeholders [115]. The understanding of One Health aspects of ASF can mobilize different disciplines and sectors to tackle health challenges [116,117]. For instance, establishing public-private partnerships between veterinary authorities and key private-sector stakeholders can be advantageous [118]. The goal, ultimately, is the translation and implementation of control measures into the local context. These need to be as simple as possible while ensuring holistic risk management in the value chain affected by ASF.

## 6. Conclusions

ASFV causes a highly infectious pig disease that affects all One Health interfaces. The virus poses a great burden on swine populations across the globe, both domestic and wild pigs, being highly lethal, particularly for naïve herds. Wildlife conservation and welfare are further hindered by preventing measures of ASF spread in wild boar, as well as threatening endangered wild pig species. Environmental linkage with disease spread and climate change are pointed out, although in-depth research is still needed. Inevitably linked to the disease is the human interface. Vast negative direct and indirect economic impacts on national industries were observed, as well as related trade of goods. Societal implications, such as existential fear of farmers, stigmatization, and public debate were observed. Here, appropriate policymaking is necessary. However, each country presents different socio-political, ecological, epidemiological, and economic settings. This leads to great variability among countries regarding disease control strategies. Prediction, prevention, mitigation, and restoration phases of ASF outbreaks require consideration of the cultural, political, industrial, economic, and psychological components of complex societies. This stresses the need for a One Health perspective on the multiple dimensions of ASF as a global infectious disease.

Taken together, ASF reflects our globalized world, with its complex biological backgrounds and complicating factors that lead to the rapid spread of ASF.

Challenges, such as economic restrictions, and possibilities, such as collaborations across nations, were highlighted. Interregional and interdisciplinary exchange is needed. Existing cooperations, guidelines, knowledge pipelines, and research approaches can be used to address ASF control gaps. Ultimately, the effort is to be put in by all involved stakeholders to mitigate the vast consequences of the disease for animals, humans, and the environment.

## Figures and Tables

**Table 1:** Summary of the ASF forms and their characteristics. Source of information: Beltran-Alcrudo, Gallardo, Kramer, Penrith, Kamata and Wiersma [10].

Form	Virulence	Mortality	Clinical Signs	Post-Mortem Findings	Comments
Peracute	High	90–100%	High fever; appetite-, and vitality loss; sudden death	Usually, no clear lesions are found	Sudden death may occur without developing clinical signs or pathological lesions
Acute	High	60–100%	Fever; inactivity; huddle behaviour; increased respiratory rate; cyanotic areas; (necrotic-) haemorrhages on ears, abdomen, and hint legs; ocular and nasal discharge; hyperaemic areas on chest, abdomen and extremities; constipation; diarrhoea (mucoid or bloody); vomiting; late abortion; death may occur after 6–15 days p.i.	Enlarged-, oedematous-, and haemorrhagic lymph nodes; hemorrhagic or hyperaemic splenomegaly; petechiae on the kidneys capsule; generalized oedemas; skin haemorrhages	Most common form; Similar signs and pathological lesions are observed in feral pigs and wild boar, although they are not so obvious due thick darker skin and fur
Subacute	Moderate	20–80%	Similar to the acute form but milder	Commonly enlarged and haemorrhagic spleen; interstitial pneumonia; congested and oedematous lung	Mainly in endemic regions; animals either die 7–20 days p.i. or recover after 30 days p.i.
Chronic	Low/ attenuated	10–30%	Mild fever; respiratory symptoms; joint swelling	Possible hyperaemic-necrotic skin areas; pneumonia with caseous-mineralized necrosis; fibrinous pericarditis; oedematous-haemorrhagic mediastinal lymph nodes	Described in regions with longer disease history, i.e., Italy, Spain, Angola
Asymptomatic	Low/attenuated host/adapted host	0%	-	-	Described especially in endemic areas [13,14,15,16].

## Data Availability

No new data were created or analyzed in this study. Data sharing is not applicable to this article.

## References

[1] World Organisation for Animal Health (WOAH) (2022). African Swine Fever (Asf)—Situation Report 14 14/06/2022.

[2] Penrith M.L., Kivaria F.M. (2022). One hundred years of African swine fever in Africa: Where have we been, where are we now, where are we going?. Transbound. Emerg. Dis..

[3] Mackenzie J.S., Jeggo M. (2019). The One Health approach—Why is it so important?. Trop. Med. Infect. Dis..

[4] Zinsstag J., Schelling E., Waltner-Toews D., Tanner M. (2011). From “one medicine” to “one health” and systemic approaches to health and well-being. Prev. Vet. Med..

[5] Zinsstag J., Crump L., Schelling E., Hattendorf J., Maidane Y.O., Ali K.O., Muhummed A., Umer A.A., Aliyi F., Nooh F. (2018). Climate change and one health. FEMS Microbiol. Lett..

[6] McEwen S.A., Collignon P.J. (2018). Antimicrobial resistance: A one health perspective. Microbiol. Spectr..

[7] Bidaisee S., Macpherson C.N. (2014). Zoonoses and one health: A review of the literature. J. Parasitol. Res..

[8] Jori F., Bastos A.D. (2009). Role of wild suids in the epidemiology of African swine fever. EcoHealth.

[9] Dixon L.K., Stahl K., Jori F., Vial L., Pfeiffer D.U. (2020). African Swine Fever Epidemiology and Control. Annu. Rev. Anim. Biosci..

[10] Beltran-Alcrudo D., Gallardo M., Kramer S., Penrith M., Kamata A., Wiersma L. (2017). African Swine Fever: Detection and Diagnosis.

[11] Schulz K., Staubach C., Blome S. (2017). African and classical swine fever: Similarities, differences and epidemiological consequences. Vet. Res..

[12] Chenais E., Depner K., Guberti V., Dietze K., Viltrop A., Ståhl K. (2019). Epidemiological considerations on African swine fever in Europe 2014–2018. Porc. Health Manag..

[13] Abworo E.O., Onzere C., Amimo J.O., Riitho V., Mwangi W., Davies J., Blome S., Bishop R.P. (2017). Detection of African swine fever virus in the tissues of asymptomatic pigs in smallholder farming systems along the Kenya–Uganda border: Implications for transmission in endemic areas and ASF surveillance in East Africa. J. Gen. Virol..

[14] Chang’A J.S., Mayenga C., Settypalli T.B.K., Achenbach J.E., Mwanandota J.J., Magidanga B., Cattoli G., Jeremiah M., Kamigwe A., Guo S. (2019). Symptomatic and asymptomatic cases of African swine fever in Tanzania. Transbound. Emerg. Dis..

[15] Patrick B.N., Machuka E.M., Githae D., Banswe G., Amimo J.O., Ongus J.R., Masembe C., Bishop R.P., Steinaa L., Djikeng A. (2020). Evidence for the presence of African swine fever virus in apparently healthy pigs in South-Kivu Province of the Democratic Republic of Congo. Vet. Microbiol..

[16] Chambaro H.M., Sasaki M., Sinkala Y., Gonzalez G., Squarre D., Fandamu P., Lubaba C., Mataa L., Shawa M., Mwape K.E. (2020). Evidence for exposure of asymptomatic domestic pigs to African swine fever virus during an inter-epidemic period in Zambia. Transbound. Emerg. Dis..

[17] Ho J., Bremang A., Conan A., Tang H., Oh Y., Pfeiffer D. (2022). Guidelines for African Swine Fever (ASF) Prevention and Control in Smallholder Pig Farming in Asia: Culling and Disposal of Pigs in an African Swine Fever Outbreak.

[18] Commission Implementing Regulation (EU) 2021/605. 7 April 2021. https://eur-lex.europa.eu/eli/reg_impl/2021/605/oj/eng.

[19] Wang T., Sun Y., Qiu H.-J. (2018). African swine fever: An unprecedented disaster and challenge to China. Infect. Dis. Poverty.

[20] Weaver T.R., Habib N. (2020). Evaluating Losses Associated with African Swine Fever in the People’s Republic of China and Neighboring Countries.

[21] Staff R. (2019). Poland’s Wild Boar Cull Prompts Protests.

[22] Television B.N. ASF: New Protests Planned Because of Compulsory Culling of Pigs 2019. https://bnt.bg/news/asf-new-protests-planned-because-of-compulsory-culling-of-pigs-236280news.html.

[23] Trotta A., Marinaro M., Cavalli A., Cordisco M., Piperis A., Buonavoglia C., Corrente M. (2022). African Swine Fever—How to Unravel Fake News in Veterinary Medicine. Animals.

[24] Essack S.Y. (2018). Environment: The neglected component of the One Health triad. Lancet Planet. Health.

[25] Cook R., Karesh W., Osofsky S. (2004). One World, One Health: Building Interdisciplinary Bridges to Health in a Globalized World.

[26] Tiwari S., Dhakal T., Kim T.-S., Lee D.-H., Jang G.-S., Oh Y. (2022). Climate Change Influences the Spread of African Swine Fever Virus. Vet. Sci..

[27] Bergmann H., Schulz K., Conraths F.J., Sauter-Louis C. (2021). A Review of Environmental Risk Factors for African Swine Fever in European Wild Boar. Animals.

[28] Capper J. Advancing environmental sustainability through better livestock health & welfare. Proceedings of the Global Forum for Food and Agriculture Expert Panel.

[29] Malogolovkin A., Yelsukova A., Gallardo C., Tsybanov S., Kolbasov D. (2012). Molecular characterization of African swine fever virus isolates originating from outbreaks in the Russian Federation between 2007 and 2011. Vet. Microbiol..

[30] Heath L., Dixon L., Sanchez–Vizcaino J. (2020). The role of ticks in the transmission and maintenance of ASF. Panor. (OIE Bull.).

[31] Luskin M.S., Meijaard E., Surya S., Walzer C., Linkie M. (2021). African Swine Fever threatens Southeast Asia’s 11 endemic wild pig species. Conserv. Lett..

[32] Pietschmann J. (2020). Afrikanische Schweinepest. Doctoral Dissertation.

[33] Wir-sind-tierarzt TVT: Keine Jagd auf Bachen mit Frischlingen Trotz ASP-Gefahr 2018. https://www.wir-sind-tierarzt.de/2018/02/tvt-stellungnahme-afrikanische-schweinepest/.

[34] Khomenko S. (2020). Challenges to wildlife management and conservation due to spread of ASF. Proceedings of the African Swine Fever: Call for Action; Series of webinars.

[35] Busch F., Haumont C., Penrith M.-L., Laddomada A., Dietze K., Globig A., Guberti V., Zani L., Depner K. (2021). Evidence-Based African Swine Fever Policies: Do We Address Virus and Host Adequately?. Front. Vet. Sci..

[36] Federal Ministry of Food and Agriculture (2021). African Swine Fever in Germany. https://food.ec.europa.eu/system/files/2021-07/reg-com_ahw_20210713_asf_deu.pdf.

[37] Kolberg S., Schulte R. (2022). Afrikanische Schweinepest: Aktuelle Erkenntnisse, Schlussfolgerungen und Forderungen. https://www.nabu.de/imperia/md/content/nabude/landwirtschaft/220818-nabu-standpunkt-afrikanische-schweinepest.pdf.

[38] Beek V.T. ASF Germany: Border Fence Criticised. Pig Progress. https://www.pigprogress.net/health-nutrition/health/asf-germany-border-fence-criticised/.

[39] (2022). Bundesforst baut Kletterhilfen für Wolf über ASP-Zaun. https://www.agrarheute.com/land-leben/rampen-ueber-asp-zaeune-kletterhilfen-fuer-wolf-co-589913.

[40] Jo Y.S., Gortázar C. (2021). African swine fever in wild boar: Assessing interventions in South Korea. Transbound. Emerg. Dis..

[41] Penrith M.-L., Bastos A., Chenais E. (2021). With or without a vaccine—A review of complementary and alternative approaches to managing african swine fever in resource-constrained smallholder settings. Vaccines.

[42] World Organization for Animal Health One Health. https://www.woah.org/en/what-we-do/global-initiatives/one-health/.

[43] Augère-Granier M.-L. (2020). The EU Pig Meat Sector. https://www.europarl.europa.eu/RegData/etudes/BRIE/2020/652044/EPRS_BRI.

[44] Bellini S. (2021). The pig sector in the European Union. Understanding and Combatting African Swine Fever: A European Perspective.

[45] Thanapongtharm W., Linard C., Chinson P., Kasemsuwan S., Visser M., Gaughan A.E., Epprech M., Robinson T.P., Gilbert M. (2016). Spatial analysis and characteristics of pig farming in Thailand. BMC Vet. Res..

[46] Huynh T., Aarnink A., Drucker A., Verstegen M. (2006). Pig production in Cambodia, Laos, Philippines, and Vietnam: A review. Asian J. Agric. Dev..

[47] Guberti V., Khomenko S., Masiulis M., Kerba S. (2019). African Swine Fever in Wild Boar Ecology and Biosecurity.

[48] Woonwong Y., Do Tien D., Thanawongnuwech R. (2020). The Future of the Pig Industry After the Introduction of African Swine Fever into Asia. Anim. Front..

[49] You S., Liu T., Zhang M., Zhao X., Dong Y., Wu B., Wang Y., Li J., Wei X., Shi B. (2021). African swine fever outbreaks in China led to gross domestic product and economic losses. Nat. Food.

[50] Casal J., Tago D., Pineda P., Tabakovski B., Santos I., Benigno C., Huynh T., Ciaravino G., Beltran-Alcrudo D. (2022). Evaluation of the economic impact of classical and African swine fever epidemics using OutCosT, a new spreadsheet-based tool. Transbound. Emerg. Dis..

[51] Brown V.R., Miller R.S., McKee S.C., Ernst K.H., Didero N.M., Maison R.M., Grady M.J., Shwiff S.A. (2021). Risks of introduction and economic consequences associated with African swine fever, classical swine fever and foot-and-mouth disease: A review of the literature. Transbound. Emerg. Dis..

[52] Pitts N., Whitnall T. (2019). Impact of African swine fever on global markets. Agric. Commod..

[53] Huang Y., Li J., Zhang J., Jin Z. (2021). Dynamical analysis of the spread of African swine fever with the live pig price in China. Math. Biosci. Eng..

[54] Hall M.J., Ng A., Ursano R.J., Holloway H., Fullerton C., Casper J. (2004). Psychological impact of the animal-human bond in disaster preparedness and response. J. Psychiatr. Pract..

[55] Mtui-Malamsha N., Assenga J., Swai E., Msemwa F., Makungu S., Chinyuka H., Bernard J., Sallu R., OleNeselle M., Ponsiano E. (2020). Subnational operationalization of one health: Lessons from the establishment of One Health rapid response teams in Tanzania. Trans. R. Soc. Trop. Med. Hyg..

[56] Wadoum R.E.G., Lichoti J.K., Nantima N., Austine B., Amara L., Sesay A., Jolo D., Conteh A., Leigh M., Marah J. (2020). Quantitative outcomes of a One Health approach to investigate the first outbreak of African swine fever in the Republic of Sierra Leone. Glob. J. Med. Re. K Interdiscip..

[57] Arias M., Sánchez-Vizcaíno J.M., Morilla A., Yoon K.-J., Zimmerman J.J. (2002). African Swine Fever. Trends in Emerging Viral Infections of Swine.

[58] Das S., Deka P., Deka P., Kalita K., Ansari T., Hazarika R., Barman N.N. (2021). African swine fever: Etiology, epidemiology, control strategies and progress toward vaccine development: A comprehensive review. J. Entomol. Zool. Stud..

[59] Gregg D.A., Mebus C.A., Schlafer D.H. (1995). African swine fever interference with foot-and-mouth disease infection and seroconversion in pigs. J. Vet. Diagn. Investig..

[60] Maté V. (1996). España Tuvo Cerradas las Fronteras Durante 30 Años Por la Peste Porcina.

[61] Raffin C. (2022). La Peste Porcina Que Tumbó el Mercado Chino se Acerca a las Distintas Granjas Españolas.

[62] Park H., Chun M.S., Joo Y. (2020). Traumatic Stress of Frontline Workers in Culling Livestock Animals in South Korea. Animals.

[63] Makita K., Tsuji A., Iki Y., Kurosawa A., Kadowaki H., Tsutsumi A., Nogami T., Watari M. (2015). Mental and physical distress of field veterinarians during and soon after the 2010 foot and mouth disease outbreak in Miyazaki, Japan. Rev. Sci. Tech..

[64] Losada-Espinosa N., Miranda-De la Lama G., Estévez-Moreno L. (2020). Stockpeople and animal welfare: Compatibilities, contradictions, and unresolved ethical dilemmas. J. Agric. Environ. Ethics.

[65] Valadez-Noriega M., Estévez-Moreno L., Rayas-Amor A., Rubio-Lozano M., Galindo F., Miranda-de la Lama G. (2018). Livestock hauliers’ attitudes, knowledge and current practices towards animal welfare, occupational wellbeing and transport risk factors: A Mexican survey. Prev. Vet. Med..

[66] Montgomery R.E. (1921). On a form of swine fever occurring in British East Africa (Kenya Colony). J. Comp. Pathol. Ther..

[67] Scott G. (1965). The virus of African swine fever and its transmission. Bull. -Off. Int. Des. Epizoot..

[68] Pini A., Hurter L. (1975). African swine fever: An epizootiological review with special reference to the South African situation. J. S. Afr. Vet. Assoc..

[69] Wilkinson P., Pensaert M. (1989). African Swine Fever Virus. Virus Infect. Porc..

[70] Plowright W., Thomson G., Neser J., Coetzer J., Thomson G., Tustin R. (1996). African Swine Fever. Infectious Diseases of Livestock, with Special Reference to Southern Africa. Aust. Vet. J..

[71] FAO (2000). African swine fever. Empress-Transbound. Anim. Dis. Bull..

[72] Manso Ribeiro J., Azevedo R., Teixeira J., Braco M., Rodrıguez A., Oliveira E., Noronha F., Grave C., Vigario J. (1963). An atypical strain of swine fever virus in Portugal. Bull. OIE.

[73] Andrade C. African swine fever in Brazil: 3 years of laboratory experience. Proceedings of the FAO/CEE Expert Consultation on African Swine Fever Research.

[74] Cwynar P., Stojkov J., Wlazlak K. (2019). African swine fever status in Europe. Viruses.

[75] Gavier-Widén D., Ståhl K., Dixon L. (2020). No hasty solutions for African swine fever. Science.

[76] Rowlands R.J., Michaud V., Heath L., Hutchings G., Oura C., Vosloo W., Dwarka R., Onashvili T., Albina E., Dixon L.K. (2008). African Swine Fever Virus Isolate, Georgia, 2007. Emerg. Infect Dis..

[77] Gogin A., Gerasimov V., Malogolovkin A., Kolbasov D. (2013). African swine fever in the North Caucasus region and the Russian Federation in years 2007–2012. Virus Res..

[78] Wang Q., Ren W., Bao J., Ge S., Li J., Li L. (2018). The first outbreak of African swine fever was confirmed in China. China Anim. Health Insp..

[79] Mighell E., Ward M.P. (2021). African Swine Fever spread across Asia, 2018–2019. Transbound. Emerg. Dis..

[80] OIE-WAHIS. https://wahis.oie.int/#/dashboards/country-or-disease-dashboard.

[81] CISA-INIA. https://eysa-cisa-inia.maps.arcgis.com/apps/MapJournal/index.html?appid=23e49a167cfb4c3fb2ab79b56b5bb742.

[82] Saker L., Lee K., Cannito B., Gilmore A., Campbell-Lendrum D.H. (2004). Globalization and Infectious Diseases: A Review of the Linkages.

[83] Anand A. (2022). China’s April Soybean Imports Slide on Year—S&P Global.

[84] Cowled M.W.B., Holley M.C., Oberin A. (2022). Hillman African Swine Fever in Wild Pigs in the Asia and the Pacific Region.

[85] Excler J.-L., Saville M., Berkley S., Kim J.H. (2021). Vaccine development for emerging infectious diseases. Nat. Med..

[86] Tatar M., Shoorekchali J.M., Faraji M.R., Wilson F.A. (2021). International COVID-19 vaccine inequality amid the pandemic: Perpetuating a global crisis?. J. Glob. Health.

[87] Muñoz-Pérez C., Jurado C., Sánchez-Vizcaíno J.M. (2021). African swine fever vaccine: Turning a dream into reality. Transbound. Emerg. Dis..

[88] Blome S., Franzke K., Beer M. (2020). African swine fever—A review of current knowledge. Virus Res..

[89] Arias M., De la Torre A., Dixon L., Gallardo C., Jori F., Laddomada A., Martins C., Parkhouse R.M., Revilla Y., Rodriguez F. (2017). Approaches and perspectives for development of African swine fever virus vaccines. Vaccines.

[90] Tran X.H., Le T.T.P., Nguyen Q.H., Do T.T., Nguyen V.D., Gay C.G., Borca M.V., Gladue D.P. (2022). African swine fever virus vaccine candidate ASFV-G-ΔI177L efficiently protects European and native pig breeds against circulating Vietnamese field strain. Transbound. Emerg. Dis..

[91] FAO, OIE (2020). Global Control of African Swine Fever: A GF-TADs Initiative 2020–2025. https://www.woah.org/app/uploads/2021/06/global-control-of-african-swine-fever-a-gf-tads-initiative-2020-2025.pdf.

[92] Kingdon J.W., Stano E. (1984). Agendas, Alternatives, and Public Policies.

[93] Stärk K.D., Regula G., Hernandez J., Knopf L., Fuchs K., Morris R.S., Davies P. (2006). Concepts for risk-based surveillance in the field of veterinary medicine and veterinary public health: Review of current approaches. BMC Health Serv. Res..

[94] Coradduzza E., Loi F., Porcu F., Mandas D., Secci F., Pisanu M.E., Pasini C., Zuddas C., Cherchi M., Denurra D. (2023). Passive Surveillance as a Key Tool to African Swine Fever Eradications in Wild Boar: A Standardized Protocol to Find the Carcasses in Mediterranean Area.

[95] Baños J.V., Boklund A., Gogin A., Gortázar C., Guberti V., Helyes G., Kantere M., Korytarova D., Linden A., EFSA (2022). Epidemiological analyses of African swine fever in the European Union: (September 2020 to August 2021). EFSA J..

[96] Barnes A.P., Moxey A.P., Ahmadi B.V., Borthwick F.A. (2015). The effect of animal health compensation on ‘positive’behaviours towards exotic disease reporting and implementing biosecurity: A review, a synthesis and a research agenda. Prev. Vet. Med..

[97] WOAH (2019). Manual of Diagnostic Tests and Vaccines for Terrestrial Animals.

[98] Brown V.R., Bevins S.N. (2018). A review of classical swine fever virus and routes of introduction into the United States and the potential for virus establishment. Front. Vet. Sci..

[99] Brown V.R., Bevins S.N. (2018). A review of African swine fever and the potential for introduction into the United States and the possibility of subsequent establishment in feral swine and native ticks. Front. Vet. Sci..

[100] Ramirez A., Whitney D., Bickett-Weddle D.A. Foreign Animal Disease Preparedness & Response Plan. https://www.academia.edu/34786380/Foreign_Animal_Disease_Preparedness_and_Response_Plan.

[101] Sykes A.L., Galvis J.A., O’Hara K.C., Corzo C., Machado G. (2023). Estimating the effectiveness of control actions on African swine fever transmission in commercial swine populations in the United States. Prev. Vet. Med..

[102] Schambow R., Colin Y., Dave W., Schettino D.N., Perez A.M. (2022). Enhancing passive surveillance for African swine fever detection on US swine farms. Front. Vet. Sci..

[103] Schettino D., Perez A., Lantigua E., Beemer O., Remmenga M., Vanicek C., Lopes G., Arzt J., Reyes R. (2023). Enhanced Passive Surveillance for Early Detection of African and Classical Swine Fevers.

[104] Bui T.T.N., Padungtod P., Depner K., Chuong V.D., Duy D.T., Anh N.D., Dietze K. (2022). Implications of partial culling on African swine fever control effectiveness in Vietnam. Front. Vet. Sci..

[105] Yoo D., Kim H., Lee J.Y., Yoo H.S. (2020). African swine fever: Etiology, epidemiological status in Korea, and perspective on control. J. Vet. Sci..

[106] Mulumba-Mfumu L.K., Saegerman C., Dixon L.K., Madimba K.C., Kazadi E., Mukalakata N.T., Oura C.A., Chenais E., Masembe C., Ståhl K. (2019). African swine fever: Update on Eastern, Central and Southern Africa. Transbound. Emerg. Dis..

[107] Penrith M.L., Bastos A.D., Etter E.M., Beltrán-Alcrudo D. (2019). Epidemiology of African swine fever in Africa today: Sylvatic cycle versus socio-economic imperatives. Transbound. Emerg. Dis..

[108] Costard S., Perez A.M., Zagmutt F.J., Pouzou J.G., Groenendaal H. (2022). Partitioning, a novel approach to mitigate the risk and impact of African Swine Fever (ASF) in endemic settings. Front. Vet. Sci..

[109] Penrith M.-L., Edoukou G. Concept Paper for Strategies for the Control, Eradication or Containment of African Swine Fever in Africa. https://agris.fao.org/search/en/providers/122621/records/6472419c2c1d629bc978f8f4.

[110] Ahmed Elsawalhy H.B., James W. Regional Strategy for the control of African Swine Fever in Africa. Proceedings of the Webinar, Food and Agriculture Organization of the UN.

[111] Schulz K., Conraths F.J., Blome S., Staubach C., Sauter-Louis C. (2019). African Swine Fever: Fast and Furious or Slow and Steady?. Viruses.

[112] Vellisco S. (1996). Erradicacion de la Peste Porcina Africana: Una Larga Historia de Esfuerzos Humanos y Materiales en la Lucha Contra Una Enfermedad Exotica.

[113] Vaclavek P. (2019). Regional ASF Wild Boar Management Workshop Belgrade, Serbia.

[114] Goethem B.V. (2020). EU’s Experience on the Prevention, Control and Eradication of African Swine Fever.

[115] Plavšic B., Rozstalnyy A., Park J., Guberti V., Depner K., Torres G. Strategic challenges to global control of African swine fever. Proceedings of the 87th General Sessions on the World Assembly of the Delegates of the OIE.

[116] Adisasmito W.B., Almuhairi S., Behravesh C.B., Bilivogui P., Bukachi S.A., Casas N., Becerra N.C., Charron D.F., Chaudhary A., Zanella J.R.C. (2022). One Health: A new definition for a sustainable and healthy future. PLoS Pathog..

[117] Barnett T., Pfeiffer D.U., Hoque M.A., Giasuddin M., Flora M.S., Biswas P.K., Debnath N., Fournié G. (2020). Practising co-production and interdisciplinarity: Challenges and implications for one health research. Prev. Vet. Med..

[118] Kim Y., Conan A., Bremang A., Tang H., Oh Y., Pfeiffer D. (2022). Guidelines for African Swine Fever (ASF) Prevention and Control in Smallholder Pig Farming in Asia: Clean Chain Approach for African Swine Fever in Smallholder Settings.

